# Effect of 2020–21 and 2021–22 Highly Pathogenic Avian Influenza H5 Epidemics on Wild Birds, the Netherlands

**DOI:** 10.3201/eid3001.230970

**Published:** 2024-01

**Authors:** Valentina Caliendo, Erik Kleyheeg, Nancy Beerens, Kees C.J. Camphuysen, Rommert Cazemier, Armin R.W. Elbers, Ron A.M. Fouchier, Leon Kelder, Thijs Kuiken, Mardik Leopold, Roy Slaterus, Marcel A.H. Spierenburg, Henk van der Jeugd, Hans Verdaat, Jolianne M. Rijks

**Affiliations:** Utrecht University, Utrecht, the Netherlands (V. Caliendo, J.M. Rijks);; Sovon, Dutch Centre for Field Ornithology, Nijmegen, the Netherlands (E. Kleyheeg, R. Slaterus);; Wageningen Bioveterinary Research, Lelystad, the Netherlands (N. Beerens);; Royal Netherlands Institute for Sea Research, Den Burg, the Netherlands (K.C.J. Camphuysen);; Wetterskip Fryslan, Leeuwarden, the Netherlands (R. Cazemier);; Wageningen Bioveterinary Research, Lelystad (A.R.W. Elbers);; Erasmus University Medical Center, Rotterdam, the Netherlands (R.A.M. Fouchier, T. Kuiken);; Staatsbosbeheer, Amersfoort, the Netherlands (L. Kelder);; Wageningen Marine Research, Den Helder, the Netherlands (M. Leopold, H. Verdaat);; Netherlands Food and Consumer Product Safety Authority, Utrecht (M.A.H. Spierenburg);; Vogeltrekstation—Dutch Centre for Avian Migration and Demography NIOO-KNAW, Wageningen, the Netherlands. (H. van der Jeugd);; Waarneming.nl, Stichting Observation International, Den Helder (H. Verdaat)

**Keywords:** influenza, avian influenza, HPAI, wild birds, H5N1, barnacle goose, sea birds, the Netherlands, viruses, respiratory infections

## Abstract

The number of highly pathogenic avian influenza (HPAI) H5-related infections and deaths of wild birds in Europe was high during October 1, 2020–September 30, 2022. To quantify deaths among wild species groups with known susceptibility for HPAI H5 during those epidemics, we collected and recorded mortality data of wild birds in the Netherlands. HPAI virus infection was reported in 51 bird species. The species with the highest numbers of reported dead and infected birds varied per epidemic year; in 2020–21, they were within the Anatidae family, in particular barnacle geese (*Branta leucopsis*) and in 2021–22, they were within the sea bird group, particularly Sandwich terns (*Thalasseus sandvicensis*) and northern gannet (*Morus bassanus*). Because of the difficulty of anticipating and modeling the future trends of HPAI among wild birds, we recommend monitoring live and dead wild birds as a tool for surveillance of the changing dynamics of HPAI.

The dynamics of highly pathogenic avian influenza (HPAI) virus infection of the H5 GS/GD lineage (clade 2.3.4.4b) have dramatically changed for wild birds. For 2 recent epidemic seasons (2020–21 and 2021–22), HPAI H5 viruses have adapted to survive long term in wild bird populations; they are now considered enzootic in wild birds (*1*–*3*). This change in status was supported by the shift in HPAI epidemiology during summer 2021, as the virus circulated continuously in northwestern Europe and Scandinavia (*1*,*4*). High rates of virus detection in wild and captive birds continued in 2022 for the largest epidemic observed to date in Europe (*4*). The circulation of HPAI virus during the 2022 breeding season exposed several colony-breeding seabird species along the northwest coast of Europe to infection (*4*–*7*), culminating in a high number of HPAI virus detections in dead wild birds during June–August 2022. At that time several seabird species exhibited widespread and massive deaths from HPAI H5N1 virus at their breeding colonies in Germany, the Netherlands, France, and the United Kingdom (*4*–*8*). Authorities have recommended reporting the number of wild birds found dead or ill during HPAI-associated dieoffs, both to contribute to the understanding of the ecologic effect of HPAI outbreaks and for targeted, evidence-based policy making (*4*,*9*).

The extent to which bird species are associated with HPAI largely depends on how often each species has tested positive (*10*). Several factors play a role in this assessment: species-specific susceptibility to clinical disease, local population size, geographic and climate circumstances, reporter effort, and number of birds screened during surveillance.

In the Netherlands, the AI-Impact working group, a consortium of ornithologists, virologists, epidemiologists, nature managers, and animal health organizations, has been active since 2020 to provide up-to-date information on wild bird mortalities during HPAI outbreaks. The aims of the consortium are to identify the range of wild bird species exposed and affected by HPAI, and to estimate the HPAI-associated level of mortality of wild birds, also relative to their population size.

For this study, we collected dead-bird reports and virologic diagnoses from a variety of sources to estimate species-specific mortality prevalence caused by the 2020–21 and 2021–22 HPAI H5 epidemics in the Netherlands. During the epidemics, mortality data were collected monthly and provided near–real-time information on the trend of the epidemic for interested organizations.

## Methods

The methods for this study were similar to those used by Kleyheeg et al. (*11*). In brief, we collected wild bird mortality data from 2 complete bird influenza seasons, October 1, 2020–September 30, 2021, and October 1, 2021–September 30, 2022. The national competent authorities reported dead wild birds to the AI-Impact consortium as part of the national animal infectious disease surveillance program and by citizen scientists who were invited to report sightings of dead birds on the web platforms of the AI-Impact members (https://dwhc.nl/dode-vogels-melden/, https://www.sovon.nl/nl/content/vogelgriep, https://www.nvwa.nl/onderwerpen/vogelgriep-preventie-en-bestrijding, https://waarneming.nl). In addition, all seabird strandings data along the Dutch North Sea and Wadden Sea coasts were analyzed as part of a long-term monitoring project and checked for unusually high numbers of stranded birds with effort correction (number per km of coastline searched, *n* km^–1^) (*12*,*13*). Unusually high densities (i.e., stranded birds per area) were >5 times background densities, as measured using identical surveys from the previous 40 years in any given month. Double counts did not occur in this dataset because carcasses were marked.

We categorized reports by bird species, date, and location of finding. Double counts (e.g., multiple entries for the same species on the same date, at the same location from the same observer) were excluded as much as possible. Consistent with similar studies, we found it highly likely that the number of reported carcasses substantially underestimates actual deaths; for example, collection rates of water bird carcasses during typical avian botulism outbreaks are 10%–25% (*14*).

We categorized wild bird mortality reports into 4 groups: Anatidae (i.e., geese, swans, ducks), other water birds (including gulls, grebes, herons, cormorants, waders, rallids), raptors, and other land birds. We classified birds of the families Podicipedidae, Laridae, Stercorariidae, Alcidae, Gaviidae, Procellariidae, Sulidae, and Phalacrocoracidae, in 1 subgroup, sea birds. We analyzed mortality data of selected species individually, because they experienced particularly high mortality rates during either the 2020–21 epidemic (i.e., barnacle goose [*Branta leucopsis*], common buzzard [*Buteo buteo*], peregrine falcon [*Falco peregrinus*], great black-backed gull [*Larus marinus*]), or during the 2021–22 epidemic (i.e., Sandwich tern [*Thalasseus sandvicensis*]*,* northern gannet [*Morus bassanus*]). We used data from the public database of Sovon (Dutch Centre for Field Ornithology, Nijmegen, the Netherlands; https://www.sovon.nl) to compare the number of reported dead wild birds per avian group during October–March (classified as winter mortality) and April–September (classified as summer mortality) between the 2020–21 and 2021–22 epidemics; we then compared data for the same months of 2010–11 with 2015–16 as described by Kleyheeg et al. (*11*). In the later period (2010–2016), there had been no outbreaks of HPAI in wild birds in the Netherlands. We tested a limited number of wild bird carcasses (Appendix 1 Table) for HPAI virus by real-time reverse transcription PCR on oropharyngeal and cloacal swabs as previously described (*15*,*16*). We submitted groups of >3 dead wild birds of certain categories (i.e., Anatidae, water birds) found dead at the same location, and single birds of other susceptible species (i.e., raptors) that were suspected of being HPAI virus-infected, for virologic analysis.

We used species data on live population estimates from the public database of Sovon to evaluate mortality rates by bird species (Table; Appendix 1 Table). Population size represents the estimated lowest and highest number of birds wintering in the Netherlands, based on census data for 2013–2020 from Sovon. For summer migratory species, population size represents the estimated lowest and highest number of birds migrating to the Netherlands, based on census data for 2016–2021 from Sovon.

**Table:** Reported bird species, nonbreeding population size estimates, number of carcasses, and RT-PCR test results for wild birds sampled during 2020–21 and 2021–22 HPAI epidemics, the Netherlands*

Avian group and species	Maximum estimated nonbreeding population size, ×1,000		No. carcasses (mortality rate, %)†			No. carcasses HPAI positive/no. tested	
			2020–21	2021–22		2020–21	2021–22
Anatidae			7,901	14,309		361/628	173/416
Geese			4,802	8,867		234/332	154/260
Barnacle goose	710–870		3,435 (1.5–4.8)	5,310 (2.4–7.4)		147/171	77/104
Graylag goose	550–670		390 (0.2–0.7)	1,054 (0.7–1.9)		30/59	53/60
Unidentified species	NA		607	1,653		35/59	36/60
Swans			996	1,453		60/136	2/17
Mute swan	41–48		305 (2.5–7.4)	479 (3.9–11)		38/93	0
Unidentified species	NA		629	969		19/54	2/17
Ducks			2,103	3,985		67/160	17/139
Eurasian wigeon	820–950		125 (<0–0.01)	300 (0.1–0.3)		12/13	1/9
Tufted duck	220–280		45 (0.6–2.5)	34 (0.01–0.1)		1/19	0
Other waterbirds			4,068	21,895		19/162	95/245
Grebes	NA		62	164		0/2	4/10
Herons	NA		250	232		0/33	3/23
Cormorants	NA		234	371		2/14	2/35
Waders	NA		1,045	1,713		9/49	10/14
Rallids	NA		327	472		0/2	1/23
Sea bird			2,371	19,340		16/102	75/140
Gulls			1,074	5,538		7/61	37/100
Great black-backed gull	25–100		137 (0.01–5.4)	372 (1.4–14.8)		1/1	1/3
Sandwich tern	27–120‡		0	5,166 (17.2–>90)§		0	29/33
Northern gannet	4–27		203	2,215 (32.8–>90)		0	6/11
Raptors			1,011	763		42/155	83/149
Common buzzard	30–50		365 (2.9–12.1)	363 (2.9–12.1)		27/91	55/81
Peregrine falcon	0.5–0.6		27 (18–54)	28 (18–56)		4/5	9/11
Other land birds			3,651	3,850		2/40	6/59
Corvids	NA		271	363		1/24	4/26
Total			16,631	41,519		427/985	357/869

*Data from Sovon (Dutch Centre for Field Ornithology, Nijmegen, the Netherlands). HPAI, highly pathogenic avian influenza; NA, not available; RT-PCR, reverse transcription PCR. †Expressed as fraction of the nonbreeding population. Lower and higher values are calculated considering the 10%–25% collection rates, as described by Kleyheeg et al. (*11*). ‡Estimated migration maximum. §Expressed as fraction of the migrant population.

## Results

A total of 16,631 wild birds of 160 species were reported dead in the Netherlands in October 1, 2020–September 30, 2021. Water birds including Anatidae accounted for 70% of the total deaths reported and land birds, including raptors, the remaining 30% (Table 1).

Anatidae by themselves represented 50% of the total deaths reported. Of the bird carcasses identified to species, by far the highest number of deaths were reported for barnacle geese (n = 3,435). The next highest numbers of dead animals were reported for graylag geese (n = 390), common buzzards (n = 365), and mute swans (*Cygnus olor*) (n = 305). HPAI virus infection was reported in 45 species (Appendix 1 Table). The species with the highest numbers of reported dead and infected birds were within the Anatidae group: barnacle geese, graylag geese, and mute swans. Common buzzard was the species with the highest numbers of reported dead and infected birds within the raptor group. Expressed as fraction of the nonbreeding population, and after accounting for detection probability, the reported dead birds represented up to 4.8% of barnacle geese, 0.7% of graylag geese, and 7.4% of mute swans (Table 1). We found the highest mortality rates occurred in raptors and scavenging species: relative to their wintering populations, up to 54% of peregrine falcons, 12.1% of common buzzards, and 5.4% of great black-backed gulls may have died.

**Figure 1:**
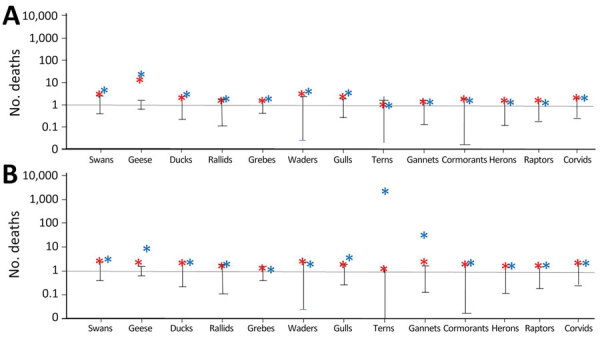
Relative number of reported deaths during the highly pathogenic avian influenza epidemics of 2020–21 (red asterisks), and 2021–22 (blue asterisks), the Netherlands. Deaths are relative to the normalized number of deaths reported over the same period from 2011–2012 to 2015–2016 (average is 1; data from Sovon, Dutch Centre for Field Ornithology, Nijmegen, the Netherlands). A) Deaths reported in the winter months, October–March. B) Deaths reported in the summer months, April–September. The y-axis is on a log scale; reported relative number of deaths among geese during winter 2021–22 was >50 larger than in the previous years. Error bars indicate maximum and minimum deaths.

A total of 41,519 wild birds of 158 species were reported dead in the Netherlands during October 1, 2021–September 30, 2022. Water birds including Anatidae accounted for 80% and land birds including raptors for the remaining 20% of the total deaths reported (Table 1). Sea birds represented >40% and Anatidae 30% of the total deaths. Of the bird carcasses identified to species, the highest number was reported for the barnacle goose (n = 5,310). The next highest numbers of dead individuals were reported for Sandwich terns (n = 5,166), and northern gannets (n = 2,215). HPAI virus infection was confirmed in 51 species (Appendix 1 Table). The species with the highest numbers of reported dead and infected birds were within the sea bird and Anatidae groups, and the species most represented were the Sandwich tern and the barnacle goose. Expressed as a fraction of the nonbreeding population, and after accounting for detection probability, the reported dead birds represented 32.8%–90% of northern gannets and up to 7.4% of barnacle geese (Table 1). The Sandwich tern appears as a summer breeder in the Netherlands; after accounting for detection probability, the reported dead birds represented 17.2%–90% of the estimated migrant population of Sandwich terns. We found that high mortality rates also occurred in raptors: up to 56% of wintering populations of peregrine falcons and 12.1% of common buzzards may have died. Mortality rates in winter or summer months were higher than the average estimates in previous years (i.e., compared to the same timeframe in 2011–2016, years in which major wild bird mortalities from outbreaks of HPAI virus did not occur). In particular, the number of reported carcasses was >50 times higher for geese in winter 2022 and >1,000 times higher for Sandwich terns in summer 2022 (Figure 1).

**Figure 2:**
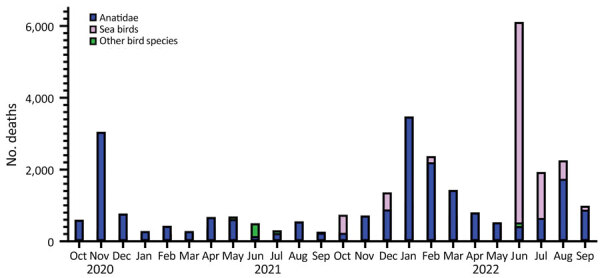
Temporal pattern of wild bird deaths during the 2020–21 and 2021–22 highly pathogenic avian influenza epidemics, the Netherlands. Sea birds include the bird families of Podicipedidae, Laridae, Stercorariidae, Alcidae, Gaviidae, Procellariidae, Sulidae, and Phalacrocoracidae.

During the 2020–21 epidemic in the Netherlands, wild bird deaths clustered in 2 peaks, the first in November 2020 and the second, smaller peak in April–May 2021 (Figure 2). During both peaks, barnacle geese were among the species most severely affected. During the 2021–22 epidemic in the Netherlands, wild bird deaths also showed 2 peaks, the first in January 2022 and the second in June 2022 (Figure 2). During the first peak, barnacle geese were again among the species most severely affected, and during the second peak, sea birds were the most severely affected. The virus was still detected in October 2022, but that date was considered the start of the new HPAI 2022–23 outbreak.

## Discussion

HPAI dynamics in wild birds are constantly evolving. The 2020–21 HPAI H5 epidemic was more devastating than earlier HPAI H5 epidemics in Europe, causing high numbers of HPAI infections and deaths in many species of wild birds (*4*,*9*,*17*–*19*). Goose species, such as the barnacle goose, accounted for the highest number of casualties. During that epidemic, high prevalence of infection in geese was also reported in Germany and United Kingdom (*2*). In our study, several duck species consistently tested positive for HPAI H5 virus during the epidemic; however, reported deaths for those species were lower than for goose species. This finding represents a different scenario than that of the 2016–17 HPAI H5 epidemic, in which duck species, such as Eurasian wigeons (*Mareca penelope*) and tufted ducks (*Aythya fuligula*), experienced the highest number of deaths (*11*). The high mortality rate of barnacle geese during the 2020–21 HPAI H5 epidemic is unprecedented. Barnacle geese are one of the most abundant geese species in the Netherlands (*19*,*20*); they are gregarious, herbivorous birds with a preference for coastal grassland and water-rich agricultural fields (*21*,*22*). Barnacle geese share their wintering habitat with other herbivorous birds, such as Eurasian wigeons and mute swans (*Cygnus olor*). The large number of geese and their gregarious behavior likely enabled the intraspecies transmission of the virus by direct or indirect contact (e.g., contaminated grass, contaminated water). The abundant circulation of HPAI H5 virus in new host species indicates that the virus has become well adapted to transmission in those species. During the 2020–21 epidemic, HPAI H5 virus was recovered from wild bird populations in the Netherlands for >1 year, indicating that it can be spread and maintained long-term in those populations (*4*,*9*), a new observation since the 2016–17 HPAI H5 epidemic, in which viral circulation was mainly limited to autumn and winter (*11*). A consequence of the unusual persistence of the virus into summer was that naive, newly hatched birds, especially juvenile Anseriformes such as mute swans and graylag geese and raptors such as white-tailed eagle (*Haliaeetus albicilla*), were exposed to the virus and died from infection during spring and summer 2021 (*9*). The large number of infected wild birds, either ill or dead, was a likely determinant for interspecies viral transmission to hunting or scavenger bird species. Raptors, for example, are exposed to infection by ingesting infected prey (*23*). During the epidemic, 11 different raptor species were found to be infected, and the highest number of infections and deaths occurred in the common buzzard (Table; Appendix 1 Table). Among the nonraptor scavenger species, we found 6 different species of gulls (Laridae) and 4 species of corvid (Corvidae) to be infected (Table; Appendix). Because the populations certain raptor species, such as the peregrine falcon, are relatively small, HPAI may represent a new threat for their conservation. Clinical signs of the infection in wild raptors were mainly neurologic, such as incoordination, body tremors, and torticollis, and were associated with brain lesions and a high level of neurotropism (*23*).

The 2021–22 HPAI H5 virus epidemic has caused the highest number of casualties among wild birds ever recorded in Europe (*4*). A significant change in the dynamic of the infections was that, since summer 2021, the virus has been enzootic in wild bird populations in Europe (*1*,*2*). This unprecedented, continuous circulation of virus during spring and summer also exposed colonial sea birds to infection (*5*). During the spring, colonial sea birds congregate in high number at their coastal breeding grounds. In this setting the virus could spread widely within and between breeding colonies, causing outbreaks that resulted in high adult and chick deaths (*6*,*7*). Sandwich terns were among the sea bird species that were more severely affected by the HPAI epidemic in the Netherlands. The sandwich tern is a vulnerable, migratory species that only breeds in a limited number of colonies across Europe seasonally, during April–September. Infection-associated mass mortality, with a mortality peak in June, was seen in 9 of the 10 Sandwich tern breeding colonies in the Netherlands (*6*). The HPAI-associated mass mortality event is a severe threat for the conservation of this species. Mass dieoffs in the breeding colonies will most likely have long-term repercussions for the Sandwich tern population (*6*). Constant monitoring of the surviving birds will be necessary to assess the long-term effect of HPAI on this species in the coming years.

The northern gannet is another colonial sea bird species that seasonally occurs off the coast of the Netherlands, although it does not breed in the Netherlands, and that was severely affected by the HPAI epidemic. The HPAI-associated infections started in April and reached a peak in July 2022. We recorded high mortality of breeding gannets on nests; large numbers of carcasses of gannets were sighted afloat near several of the largest or most important breeding colonies and widespread in the North Sea basin (*7*). The ecology and pattern of mortalities of northern gannets have been studied in the Netherlands since 1980. Data from this long-term study enabled accurate evaluation of the 2022 mortality event in relation to background mortality and corrected per observer effort (*24*). For the northern gannet the number of reported corpses in July 2022 was 66 times larger than average in previous years, the highest spike in deaths over the past 40 years (*24*).

During the 2021–22 epidemic, high mortality rates in sea bird species were also reported in other countries in and outside of Europe. For example, Dalmatian pelicans (*Pelecanus crispus*) in Greece and great skuas (*Stercorarius skua*) on Foula, United Kingdom, both had 60%–70% declines of their populations because of HPAI virus infection during colonial breeding (*7*,*8*,*25*). The high density of birds and their close contact during colonial breeding probably enabled the rapid spread of the infection within the colonies. Field data suggest that HPAI-positive birds could shed virus for some period before death, providing opportunities for direct bird-to-bird or environmental transmission (*26*). Bird species such as great skuas have been reported to bathe and socialize at freshwater lochs and pools, providing possible opportunities for conspecific infection (*7*). Scavenging activities are another possible source of infection. Unattended chicks from dead parents most likely died because of lack of parental care. Maternal antibodies have been described in chicks of previously infected parents, but clinical protection is short-lived and requires high maternal antibody titers (*27*,*28*). Furthermore, maternal antibodies are only relevant if the infection has occurred before egg laying. Infected birds of certain Anatidae species can survive HPAI virus infection with limited clinical consequences (*29*,*30*). Experimentally serially infected ducks can develop a long-term immunity that confers protection from subsequent HPAI virus infection (*29*). It is possible that sea bird species will also develop flock immunity protective at future reinfection. The surviving birds should be tested for the presence of serum antibodies to gather data about flock immunity over the next several years.

The massive number of dead birds at colonies posed a biosecurity issue through the risk for viral spillover; cleaning up was an overwhelming task for the involved authorities. The AI-Impact consortium, together with the competent health authorities, provided a decision tree for the cleanup of dead birds to reduce the environmental contamination with minimal disturbance for the remaining birds (Appendix 2; Appendix 3). Carcass removal is necessary to reduce the amount of infected material that could spread the infection in the environment (*6*). Thus, we recommend controlled studies to optimize carcass removal.

During spring 2021, for the first time since the 2005–06 HPAI H5 epidemic, the virus was detected in Europe in several carnivore species, European foxes (*Vulpes vulpes*), gray seals (*Halichoerus grypus*), and harbor seals (*Phoca vitulina*); they were most likely infected through contact with or ingestion of infected wild birds (*9*,*31*). HPAI H5 viruses were once again detected in wild mammal species in Europe during the 2021–22 season and showed genetic markers of adaptation to replication in mammals (*16*). Therefore, we recommend planned year-round active and passive surveillance of wild mammals. The zoonotic risk for infection for humans of this particular H5 virus strain is considered low for the general population and low to medium for occupationally exposed workers, such as culling operators, wild animal rehabilitators, and workers involved in carcass removal (*16*). Persons at occupational risk should wear adequate personal protective equipment and be immunized with preventive annual vaccination against human influenza to avoid reassortment with HPAI H5 virus. In case of potential infection, those persons should be monitored for respiratory symptoms, neurologic symptoms, or conjunctivitis for 10 days after exposure (*16*), and diagnostic testing, if necessary, should be conducted at the competent national health authority.

Since the end of 2016, mass mortality events among wild birds caused by HPAI H5 infection in the Netherlands have occurred in various species in various years, including Eurasian wigeon (2016), tufted duck (2016), barnacle goose (2020–2022), Sandwich tern (2022), and black-headed gull (*Chroicocephalus ridibundus*) (2023) (*16*). One characteristic those species have in common is that they live in dense groups at certain times of the year (*10*) and live close to open water. We suspected that this combination is an important risk factor for infection, because such groups have more opportunities for virus exposure and transmission and for possible species-specific adaptation of the virus (*10*). However, susceptibility to disease from HPAI virus infection seems to vary enormously between species. For example, disease and death can peak in one species while other species similarly present in the same area show hardly any signs of disease (*10*).

Because it remains difficult to anticipate and to model the future trends of HPAI among wild birds, we recommend constant monitoring of live and dead wild birds as an essential tool for surveillance of the evolving dynamics of HPAI. This method has several limitations; one is that it is difficult to exclude double-counted reports. Another is that not all the reported dead birds can be tested for HPAI virus infection, and not all will have died from HPAI infection. Two main improvements that we propose for HPAI surveillance in wild birds are long-term monitoring of HPAI-associated wild bird deaths, corrected for observer effort, and testing apparently healthy wild birds, particularly candidate reservoir species, for both HPAI virus and antibodies to HPAI virus. For the constant monitoring of wild bird deaths in the Netherlands during the 2020–21 and 2021–22 HPAI H5 epidemics, citizen scientists were a fundamental resource and made it possible to obtain a wider impression of the actual scale of mortality in wild birds, which otherwise would have been limited to the data from official surveillance. In addition to surveillance for HPAI, we recommend recording of wild bird deaths and encouraging and systematically endorsing the work of citizen scientists and international citizen-science platforms.

**Appendix 1.** Additional information about the effects of the highly pathogenic avian influenza epidemics of 2020–21 and 2021–22 on wild birds, the Netherlands.

**Appendix 2.** Information about removal of wild bird carcasses during the highly pathogenic avian influenza epidemic, 2020–21, the Netherlands.

**Appendix 3.** Information about removal of wild bird carcasses during the highly pathogenic avian influenza epidemic, 2021–22, the Netherlands.
