# Supplementary material for: Effect of 2020–21 and 2021–22 Highly Pathogenic Avian Influenza H5 Epidemics on Wild Birds, the Netherlands
Source: Emerg Infect Dis. 2024 Jan;30(1):50–7. doi: 10.3201/eid3001.230970 (PMC10756359; doi:10.3201/eid3001.230970)
Supplement: Supplementary file 2 — Appendix 2. Information about removal of wild bird carcasses during the highly pathogenic avian influenza epidemic, 2020–21, the Netherlands. [file 23-0970-Techapp-s2.pdf]

Article DOI: http://doi.org/10.3201/eid3001.230970

Effect of 2020–21 and 2021–22 Highly Pathogenic Avian Influenza H5 Epidemics on Wild Birds, the Netherlands

Appendix 2

ADVICE FOR CLEARING WILD BIRD CARCASES IN OUTDOOR AREAS DURING THE HIGH PATHOGENIC BIRD FLU OUTBREAK H5NX 2020–2021

Working group AImpact2021, 13 November 2020

Reason for advice

The first step in finding dead wild birds is reporting through the appropriate official channels: NVWA (045–546 31 88) on >3 ducks, geese, swans in (more or less) the same location, or >20 birds from other bird species in (more or less) the same location; in other cases Sovon (www.sovon.nl/nl/content/vogel-en-zoogdierdoelen) and DWHC (www.dwhc.nl/meldsvorm/). The reports together give these organizations a picture of the course of the outbreak.

In addition, the NVWA and DWHC can decide whether it is necessary to collect carcasses for bird flu diagnosis by WBVR in Lelystad. This keeps people informed of the locations and sources of infection of bird flu.

Wild bird carcasses are cleaned up in populated areas and in outlying areas where a lot of public comes, for reasons of hygiene and the risk of the virus spreading. However, it is not so clear to site managers what the best course of action is to deal with wild bird carcasses in remote

areas where there is little public access. This advice is about the latter: the choice for or against clearing wild bird carcasses in rural areas with little public.

Relevant information about highly pathogenic bird flu virus

• In general, wild birds that live in or around water (waterfowl in an ecologic sense) can be infected with avian influenza virus (AIV) through direct contact with other infected birds, or through indirect contact with environmental material (eg water, mud, grass) that is contaminated with contaminated excrement (eg faeces, snot) (*1*). Waterfowl that have often been found dead in recent years with highly pathogenic avian influenza virus (HPAIV) are ducks, geese, swans, grebes, storks, herons, cormorants, coots, waders, and gulls (*2*).

• Some bird species can become infected by eating the muscles and organs of other birds with HPAIV infection, either because they prey on infected birds or because they eat carcasses of dead, infected birds (*3*–*5*). Birds of prey and scavengers that have often been found dead with HPAIV infection in recent years include white-tailed eagle, rough-legged hawk, buzzard, peregrine falcon, hawk, short-eared owl, great black-backed gull, herring gull and magpie (*2*) (www.oie.int).

- The full list of 68 bird species in which HPAIV has been detected during the major HPAIV outbreak of 2016/2017 can be found in Table A.3 of the relevant EFSA report (*6*). Only virus positive dead birds identified by species are included.
- AIV in surface waters of the wetlands of Alaska and Minnesota remained infectious to birds for more than 7 months during the winter months (September to April) (*7*).
- In chicken carcasses experimentally infected with HPAIV, the maximum survival times for infectious virus under laboratory conditions depended on temperature. At +4°C maximum survival was 8 months in feathers, 5 months in muscle and 3 weeks in liver. At +20°C, maximum survival was 1 month in feathers, 3 weeks in muscle and 3 days in liver (*8*).
- RIVM estimates that there is a low risk of people becoming infected with the HPAIV strains that have been found in wild birds and poultry farming in recent

weeks (https://www.rijksoverheid.nl/documents/kamerstukken/2020/11/ 10/room letter update bird flu-3rd infection and low zoonosis risk).

Considerations for Disposing of Wild Bird Carcasses in Outlying Areas

- Carcasses of dead wild birds infected with HPAIV are a source of virus contamination for their environment. As a result, birds that rest or forage in that environment run an extra risk of becoming infected.
- Birds of prey and carrion-eating birds that eat HPAIV-infected carcasses are at extra risk of becoming infected.

Choice for cleaning up

- Outlying area is regularly used by raptors and carrion-eating bird species.
- Carcasses are located in outlying areas where birds rest or forage in high density (eg high water refuge, wetland area, pasture, puddle, lake).

Choice against cleaning up

- Carcasses are located in outlying areas where birds of prey or carrion-eating bird species are not or sporadically present (unsuitable habitat) or cannot easily reach (e.g. undergrowth).
- Carcasses are located in rural areas where waterfowl occur sporadically.
- There is no clear indication of carcasses, while entering the area would disturb large numbers of wild birds.

How many carcasses to clean up?

There is no specific number to indicate here. It depends on the location and the circumstances. The best advice is to clean up when there are more carcasses than normal and when they are found in situations such as those mentioned under 'Clearance choice'.

How often do you clean up carcasses?

No specific frequency can be specified here either. It depends on the course of the outbreak, and specifically the number of birds that die per day. This can best be determined by regularly (preferably daily) checking the area from a distance, and letting the frequency of clearing depend on the degree of bird death.

How to clean up?

- Enter the area with the minimum number of people needed to clean up quickly and efficiently, with as little disturbance as possible to the birds present, as this could be counterproductive. Clear high water refuges during low water to limit disturbance.
- Combine carcass removal with registration of bird deaths in the area (number per species via Sovon or DWHC Web sites. Waarneming.nl can also be used by people who are familiar with it. Preferably keep the person in charge of the registration 'clean', i.e., he refrains from direct contact with dead birds. Please report any rings present via www.vogeltrekstation.nl
- Coordinate entry into the area with an ornithologist or area manager known locally with bird knowledge (also for the registration of numbers, species and rings).
- Wear adequate personal protection, and adequate disinfection afterwards (including footwear!) to prevent contamination of other areas.
- Transport carcasses in double plastic bags to the appropriate place, to be transported to a rendering company.
- For specific details about personal protection and other hygiene measures, see: https://www.nvwa.nl/onderwerpen/vogelgriep-preventiveness-en-control/documents/dier/dierspreiden/vogelgriep/protocols/handbook-voor-het-opruiming -found-dead-wild-waterfowl.

Composition of the AImpact2021 Working Group (13 November 2020)

Valentina Caliendo (Erasmus MC), Kees Camphuysen (NIOZ), Armin Elbers (WBVR), Koos Hartnack (RWS), Leon Kelder (SBB), Erik Kleyheeg (Sovon), Thijs Kuiken (Erasmus MC, chairman), Mardik Leopold (Wageningen Marine Research), Jolianne Rijks (DWHC), Timo Roeke (waarneming.nl), Roy Slaterus (Sovon), Marcel Spierenburg (NVWA), Henk van der Jeugd (NIOO), Peter van Tulden (WBVR), Hans Verdaat (Wageningen Marine Research).

References

1. Olsen B, Munster VJ, Wallensten A, Waldenström J, Osterhaus AD, Fouchier RA. Global patterns of influenza a virus in wild birds. Science. 2006;312:384–8. PubMed https://doi.org/10.1126/science.1122438

2. Brouwer A, Gonzales J, Huneau A, Mulatti P, Kuiken T, Staubach C, et al.; European Food Safety Authority (EFSA). Annual Report on surveillance for avian influenza in poultry and wild birds in Member States of the European Union in 2018. EFSA J. 2019;17:e05945. PubMed

3. Brown JD, Stallknecht DE, Swayne DE. Experimental infections of herring gulls (Larus argentatus) with H5N1 highly pathogenic avian influenza viruses by intranasal inoculation of virus and ingestion of virus-infected chicken meat. Avian Pathol. 2008;37:393–7. PubMed https://doi.org/10.1080/03079450802216595

4. van den Brand JM, Krone O, Wolf PU, van de Bildt MW, van Amerongen G, Osterhaus AD, et al. Host-specific exposure and fatal neurologic disease in wild raptors from highly pathogenic avian influenza virus H5N1 during the 2006 outbreak in Germany. Vet Res (Faisalabad). 2015;46:24. PubMed https://doi.org/10.1186/s13567-015-0148-5

5. Krone O, Globig A, Ulrich R, Harder T, Schinköthe J, Herrmann C, et al. White-Tailed Sea Eagle (*Haliaeetus albicilla*) Die-Off Due to Infection with Highly Pathogenic Avian Influenza Virus, Subtype H5N8, in Germany. Viruses. 2018;10:478. PubMed https://doi.org/10.3390/v10090478

6. Brown I, Mulatti P, Smietanka K, Staubach C, Willeberg P, Adlhoch C, et al.; European Food Safety Authority; European Centre for Disease Prevention and Control; European Union Reference Laboratory for Avian influenza. Avian influenza overview October 2016-August 2017. EFSA J. 2017;15:e05018. PubMed

7. Ramey AM, Reeves AB, Drexler JZ, Ackerman JT, De La Cruz S, Lang AS, et al. Influenza A viruses remain infectious for more than seven months in northern wetlands of North America. Proc Biol Sci. 2020;287:20201680. PubMed https://doi.org/10.1098/rspb.2020.1680

8. Yamamoto Y, Nakamura K, Mase M. Survival of Highly Pathogenic Avian Influenza H5N1 Virus in Tissues Derived from Experimentally Infected Chickens. Appl Environ Microbiol. 2017;83:e00604–17. PubMed https://doi.org/10.1128/AEM.00604-17
