# Supplementary material for: Effect of 2020–21 and 2021–22 Highly Pathogenic Avian Influenza H5 Epidemics on Wild Birds, the Netherlands
Source: Emerg Infect Dis. 2024 Jan;30(1):50–7. doi: 10.3201/eid3001.230970 (PMC10756359; doi:10.3201/eid3001.230970)
Supplement: Supplementary file 3 — Appendix 3. Information about removal of wild bird carcasses during the highly pathogenic avian influenza epidemic, 2021–22, the Netherlands. [file 23-0970-Techapp-s3.pdf]

Article DOI: http://doi.org/10.3201/eid3001.230970

Effect of 2020–21 and 2021–22 Highly Pathogenic Avian Influenza H5 Epidemics on Wild Birds, the Netherlands

Appendix 3

ADVICE FOR REGISTRATION AND DISPOSAL OF WILD BIRD CARCASSES IN BREEDING COLONIES DURING HIGHLY PATHOGENIC AVIAN INFLUENZA OUTBREAK H5NX, 2022

Working group AI-Impact 21/22, 29 June 2022

In 2022, infection with highly pathogenic avian influenza virus H5 of the Goose/Guangdong lineage (further referred to as HPAI) more often has led to extensive mortality in breeding colonies of wild birds, including sandwich and common terns in the Netherlands, and northern gannets, great skuas and common guillemots in the UK For this reason, we have written a protocol specifically aimed at HPAI in breeding colonies. It is important to realize that while all listed birds are ‘colonial’, breeding conditions differ from high densities on sand flats (e.g., terns), via relatively low densities (territories) on hill slopes (e.g., skuas), to high densities on steep cliffs or concentrations on flat tops of remote islands (e.g., gannets and auks).

The general principle is to leave wild birds with HPAI alone, so that the virus is not spread to other areas via disturbed birds. Two important reasons to visit a breeding colony where HPAI occurs are: 1, to limit virus spread in the breeding colony by removing carcasses from sites where infections may occur (colony itself, or bathing places for exampleI); 2, to record scientific information about the outbreak, and 3. by removing carcasses from sites where they pose a risk for the general public or livestock. Below we give more details about the first two reasons.

(1) *Limiting virus spread in the breeding colony by removing carcasses*

Carcasses in a breeding colony can be a source of infection for other birds in the colony. A bird can be infected by touching the carcass (e.g., out of curiosity, seen in juvenile cormorants and pelicans; mating behavior, seen in Sandwich terns), by eating at the carcass (seen in great skuas), or by drinking water or bathing in water containing carcasses (seen in cormorants and great skuas). Avian influenza virus in carcasses and surface water can remain contagious for days to months, depending on environmental factors including the ambient temperature.

(2) *Recording information about a HPAI outbreak in a breeding colony*

In the current surveillance systems for HPAI in wild birds, the detection of HPAI virus in the event of mortality of wild birds is recorded, but not the number of wild birds found dead. Well-documented descriptions of HPAI outbreaks in wild birds are therefore important to better assess the impact of this disease on wild bird populations, and to help policymakers in making decisions to prevent future HPAI outbreaks, not only in poultry and humans, but also in wild birds.

Decision to visit a breeding colony

The final decision whether to visit a breeding colony should be taken depending on the local situation and in consultation with people with good knowledge of the breeding colony, and taking into consideration the following advantages and disadvantages:

Advantages

- removal of carcasses that may remain contagious for days to months
- more detailed recording of the outbreak than possible from a distance.

Disadvantages

- disturbance of birds, with a chance of virus being spread to other areas and/or chance that birds abandon the breeding colony
- (very small) risk of infection of people who visit the breeding colony.

Relevant information and samples to collect during a visit

To determine the extent of the outbreak, record:

- bird species
- age (external plumage characteristics)
- sex (external plumage characteristics)
- any ring numbers
- photos of sick and dead birds as supporting evidence.

To substantiate the cause of the outbreak:

- describe and/or record on film the clinical signs in affected birds.

Hygiene measures

The following hygiene measures are relevant if you choose to visit a breeding colony where HPAI occurs:

- Wear adequate personal protection, and perform adequate disinfection afterwards (including footwear!) to prevent contamination of other areas.
- Suitable disinfectants for HPAI virus include agents based on ethanol (e.g., Sterillium) or sodium hypochlorite (e.g., Clorox).
- Transport carcasses in double plastic bags to the place designated by the relevant authorities for further disposal
- For specific details on personal protection and other hygiene measures, see Web sites of the relevant authorities.

Composition of the AI Impact Working Group 21/22 (29 June 2022)

Nancy Beerens (WBVR), Valentina Caliendo (Erasmus MC), Kees Camphuysen (NIOZ), Rommert Cazemier (Wetterskip Fryslân), Armin Elbers (WBVR), Ron Fouchier (Erasmus MC), Jan Jelle Jongsma (Fryske Gea), Fedde Jonkman (Zuiderzeeland), Leon Kelder (SBB), Erik Kleyheeg (Sovon), Thijs Kuiken (Erasmus MC, chairman), Mardik Leopold (Wageningen Marine Research), Jolianne Rijks (DWHC), Ramon ter Schegget (RWS), Roy Slaterus (Sovon), Marcel Spierenburg (NVWA), Henk van der Jeugd (NIOO), Peter van Tulden (WBVR), Hans Verdaat (Wageningen Marine Research).
